# A Pathological Analysis of Canaliculitis Concretions: More Than Just* Actinomyces*

**DOI:** 10.1155/2016/6313070

**Published:** 2016-06-14

**Authors:** Balaji Perumal, John Andrew Carlson, Dale Robert Meyer

**Affiliations:** ^1^Lions Eye Institute, Albany Medical Center, 1220 New Scotland Road, Slingerlands, NY 12159, USA; ^2^Department of Pathology, Albany Medical Center, 47 New Scotland Avenue, Albany, NY 12208, USA

## Abstract

*Purpose.* Canaliculitis is classically associated with* Actinomyces* species, which are filamentous bacteria; the purpose of this study was to evaluate the extent to which nonfilamentous bacteria colonize canalicular concretions by using graded histopathological analysis.* Methods.* This is a series of 16 cases. The percentage of Gram-positive/Gomori's methenamine silver-positive filamentous bacteria (*Actinomyces*) versus the total bacteria identified was graded, and the types of bacteria seen were recorded. Nonfilamentous bacteria were categorized based upon Gram stain (positive or negative) and morphology (cocci or rods).* Results*. There were 11 females and 5 males. Nonfilamentous bacteria were identified in 16 of 16 (100%) specimens and filamentous bacteria were identified in 15 of 16 (94%) specimens. The mean percentage of filamentous bacteria relative to total bacteria was 57%. Regarding the nonfilamentous bacteria present, 69% of specimens had Gram-positive cocci only, 25% had Gram-positive and Gram-negative cocci, and 6% had Gram-positive cocci and Gram-positive rods.* Conclusion*. In the current study, there was a mix of filamentous and nonfilamentous bacteria in almost all canalicular concretions analyzed. Nonfilamentous bacteria may contribute to the pathogenesis of canaliculitis. In addition, the success of bacterial culture can be variable; therefore, pathological analysis can assist in determining the etiology.

## 1. Introduction

Canaliculitis is a relatively rare disorder which has classically been associated with* Actinomyces* species. The typical histology consists of microscopic granules with basophilic masses, an eosinophilic periphery, and a background of Gram-positive filamentous bacteria [1–3]. Repp and coworkers demonstrated that* Actinomyces* was identified on histopathology in nearly all canaliculitis concretion specimens, with infrequent identification of nonfilamentous bacteria [4]. Culture results have suggested that other Gram-positive and/or Gram-negative bacteria can be found in canaliculitis specimens [1–6]. Interestingly, a recent histopathological study by Perry and coworkers noted that Gram-positive cocci were “frequently seen colonizing the outer perimeter of the concretions,” although* Actinomyces* was the predominant organism [3]. Bacterial culture has variable success in this setting, and several studies have emphasized the utility of histopathological analysis for determining the bacterial composition of canalicular concretions [7, 8]. The purpose of this study was to determine the extent to which nonfilamentous bacteria colonize canalicular concretions by quantifying the types and proportions of bacteria present with graded histopathological analysis. This may assist in determining the etiology of canaliculitis.

## 2. Methods

A retrospective review was performed on all canalicular concretion specimens submitted for pathological analysis at Albany Medical Center between 2006 and 2013. The specimens were identified by searching the pathology database with the terms “canaliculitis” and/or “infectious granules.” Information was recorded from the pathology reports including eyelid involved, patient age, and gender. Institutional Review Board (IRB) approval was obtained for this study and it is compliant with the Health Insurance Portability and Accountability Act (HIPAA).

Canaliculitis specimens were obtained from patients through curettage or vertical canaliculotomy with retrograde expression of concretions [9]. All specimens were stained with hematoxylin and eosin (H&E) as well as Gomori's methenamine silver (GMS) and Gram stains. The GMS stain facilitated identification of filamentous bacteria such as* Actinomyces* species and Gram stain was used for the identification of other bacteria. Concretions were initially identified at low power (100x) on H&E stain. The slides were analyzed to determine how many bacteria were present as well as types of bacteria that could be identified. Upon analysis, the total number of bacteria was first estimated. This was achieved by examining each slide at 400x magnification using a 10 mm by 10 mm grid reticle (total of 100 1 mm by 1 mm individual blocks). The average number of bacteria per individual block within the grid was recorded. The relative amount of bacteria in each specimen was categorized as none (0 per individual block), “low” (1–20), “medium” (21–40), or “high” (greater than 40).

Following this analysis, the types of bacteria present were determined using 200x and 400x magnification. The proportional amount of Gram-positive/GMS-positive filamentous bacteria (*Actinomyces*) present in each specimen was then graded as a percentile of the total bacteria identified. Percentages were recorded to the nearest 5%. Nonfilamentous bacteria were recorded as Gram-positive cocci, Gram-negative cocci, Gram-positive rods, and/or Gram-negative rods based upon their morphology and Gram stain characteristics.

The English medical literature was reviewed using the search terms “canaliculitis,” “canalicular concretions,” and “lacrimal concretions.”

## 3. Results

A total of 16 specimens from 16 patients were identified which met the criteria for canaliculitis concretions. There were 11 females and 5 males. The average patient age was 61 years (range 30 to 85 years).  Table 1 outlines the demographic data and analysis for all 16 pathological specimens.

All of the specimens were found to consist of concretions on H&E stain, and all specimens contained at least some bacteria on Gram and GMS stains. When determining the grade for the total number of bacteria present in a given specimen, 6 (38%) were graded as “low,” 5 (31%) “medium,” and 5 (31%) “high.”

**Figure 1:**
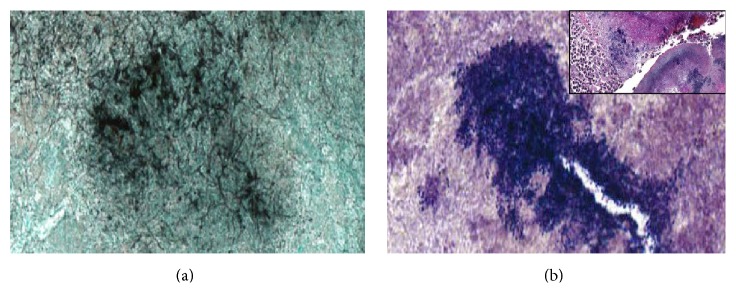
(a) Filamentous bacteria within a canalicular concretion (400x, Gomori's methenamine silver (GMS) stain). (b) Multiple Gram-positive bacteria within the same concretion (400x, Gram stain); (b) Inset: Splendore Hoeppli phenomenon with blue-staining bacteria surrounded by eosinophilic deposits (200x, hematoxylin and eosin (H&E) stain).

All 16 specimens had evidence of Gram-positive cocci; in 11 of 16 (69%) cases, the nonfilamentous bacteria were identified as Gram-positive cocci only; 4 of 16 (25%) specimens had Gram-positive and Gram-negative cocci; and 1 of 16 (6%) specimens had Gram-positive cocci and Gram-positive rods. Fifteen of sixteen specimens had filamentous bacteria. The mean percentage of filamentous bacteria relative to total bacteria was 57% (range 0 to 95%). In addition, all specimens demonstrated blue-staining bacteria surrounded by an eosinophilic fibrillary coat on H&E stain.  Table 1 provides details on the percentage of filamentous bacteria and types of bacteria identified for all of the specimens analyzed. Figures 1(a) and 1(b) illustrate examples of the histopathological findings.

## 4. Discussion

Canaliculitis is a disease which is most commonly thought to be associated with yellow, gritty concretions secondary to* Actinomyces* species. As mentioned previously, the classic histopathology in* Actinomyces-*related infection reveals basophilic masses with an eosinophilic periphery and a background of Gram-positive, filamentous bacteria [1–3]. Other organisms have also been isolated through culture in cases of canaliculitis [2, 5, 6]. Several studies have reported that* Staphylococcus* species have been cultured from the expressed material in the canaliculus. Anand and coworkers and Vecsei and coworkers reported that* Staphylococcus aureus* was the most common organism identified and cultured in 26.6% and 25.3%, respectively, of patients with canaliculitis [5, 6]. Zaldivar and Bradley found that 21% of cultures grew* Streptococcus* and 13% grew Staphylococcus [10]. Although a number of bacteria have been cultured, the success of bacterial culture in the setting of canaliculitis can be somewhat variable. Previous studies have reported positive bacterial culture rates of 27% to 91% [7, 8]. In the current study, bacteria were readily identified on pathological analysis in all concretion specimens. Given the variability in culture results, histopathological analysis can be a useful adjunct in determining the etiology in these patients.

In a recent study using histopathologic evaluation, Perry and coworkers noted the presence of frequent Gram-positive cocci in canaliculitis concretions, although the dominant organism was reported to be* Actinomyces* species. The authors of this study commented that the Gram-positive cocci may contribute to the purulent nature of canaliculitis but felt that* Actinomyces* was the predominant etiologic agent leading to infection [3]. The results of our study, reported herein, demonstrated that a mixture of bacteria in canalicular concretions is quite common, being identified in all specimens analyzed. In addition, the current study grades the amount and types of bacteria present. On average, nonfilamentous bacteria were noted to be almost half of the organisms identified in concretions. Gram-positive cocci were noted in all specimens. In contrast to the study by Perry and coworkers, 25% of samples were also found to have Gram-negative bacteria [3].

*Actinomyces* are well known to form concretions. However, other Gram-negative and Gram-positive bacteria are also known to form concretions in other parts of the body, a finding sometimes referred to as “botryomycosis.” One of the main pathological characteristics of this finding is blue-staining bacteria surrounded by an eosinophilic fibrillary coat, known as the Splendore Hoeppli phenomenon [11, 12]. This specific finding was identified in all specimens of the current study. The diagnosis of botryomycosis is often unrecognized because it can appear similar to actinomycosis both clinically and histopathologically. The most common bacteria associated with this entity are* Staphylococcus* species [13, 14]. It is thought that the organisms form granules as a defense mechanism against the body's immune system which allows them to promote the disease process. The prevalence of the Splendore Hoeppli phenomenon lends evidence to the presence of nonfilamentous bacteria in canaliculitis specimens, though the authors do not necessarily believe that it discounts the presence of* Actinomyces*. Both* Actinomyces* and nonfilamentous bacteria were found in the majority of specimens of this study, and it is plausible that they both contribute to the pathogenesis of canaliculitis. Additional research, such as polymerase chain reaction (PCR) study of canaliculitis specimens, could help to further elucidate the bacterial diversity in this disease.

Most of the nonfilamentous bacteria identified in the current study were Gram-positive cocci.* Staphylococcus* and* Streptococcus* species are included in this category and are the types identified most often by culture in prior studies [5, 6, 10]. Gram-negative cocci and Gram-positive rods were found as well, though less frequently. Previous culture studies from patients with canaliculitis have identified* Haemophilus influenza,* which can be seen as Gram-negative cocci, and Gram-positive rods such as* Corynebacterium* and* Propionibacterium* [2, 15].* Moraxella* is another Gram-negative coccus which is known to cause other ocular infections such as keratitis [16]. In addition, Gram-negative organisms have been identified in normal conjunctiva and in chronic dacryocystitis. In one study, Gram-negative bacteria were found in 20% of dacryocystitis cultures [17, 18].

The demographic characteristics of the patients included in this study were similar to previous reports, with the lower eyelids being more commonly involved (15 of 16 eyelids), the subjects were primarily female, and the average age was 61 years [19–22]. The limitations of this study include its retrospective nature and limited sample size. Also certain patient historical information, such as punctal plug placement, was not available for this study. This information could add additional information in determining the etiology of canaliculitis. While no claim is made of the superiority of histopathological analysis over bacterial culture for the evaluation of canaliculitis and associated concretions, pathological analysis can be a useful adjunct. The current study found that a mixture of bacteria (“mixed infection”) is often present in histopathologic sections of canalicular concretions and further revealed that a relatively large percentage of nonfilamentous bacteria (both Gram-positive and Gram-negative) can be seen on pathological analysis. These bacteria may contribute to the pathogenesis of canaliculitis.

## Figures and Tables

**Table 1:** Demographic data and histopathological analysis results.

Age	Gender	Eyelid	Total bacterial count	% *Actinomyces* within concretions	Nonfilamentous bacteria identified
52	M	LUL	Medium	90	Gram+ cocci
34	F	RLL	Low	0	Gram+, gram− cocci
62	F	RLL	High	95	Gram+ cocci
54	F	RLL	Medium	50	Gram+ cocci
45	M	LLL	Medium	80	Gram+, gram− cocci
69	F	LLL	Low	70	Gram+ cocci
54	F	LLL	Medium	80	Gram+ cocci
66	M	LLL	Low	40	Gram+ cocci
78	F	RLL	Low	5	Gram+ cocci
30	F	RLL	Low	50	Gram+ cocci
45	M	LLL	High	50	Gram+ cocci
65	F	RLL	High	95	Gram+ cocci
75	M	LLL	High	80	Gram+, gram− cocci
76	F	RLL	Low	20	Gram+, gram− cocci
82	F	RLL	Medium	10	Gram+ cocci, gram+ rods
85	F	RLL	High	95	Gram+ cocci

Demographic and pathological analysis data for all specimens

M: male.

F: female.

RUL: right upper lid.

RLL: right lower lid.

LLL: left lower lid.
